# Effect of simulator fidelity on skill acquisition and trainee satisfaction in arthroscopic surgery training for novices: a prospective randomized comparative study

**DOI:** 10.1007/s11548-025-03528-5

**Published:** 2025-10-09

**Authors:** Tatsuhiko Kutsuna, Tomofumi Kinoshita, Kazunori Hino, Kunihiko Watamori, Takashi Tsuda, Shintaro Yamaoka, Masaki Takao

**Affiliations:** 1 Department of Orthopaedic Surgery, Ehime University Graduate School of Medicine, 454 Shitsukawa, Toon, Ehime 791-0295 Japan; 2 Department of Joint Reconstruction, Ehime University Graduate School of Medicine, 454 Shitsukawa, Toon, Ehime 791-0295 Japan

**Keywords:** Arthroscopic surgery, Simulation, Fidelity, Trainee satisfaction, Skill acquisition

## Abstract

**Purpose:**

In recent years, simulator-based training has gained attention as a safe and effective approach for surgical education. In orthopedic surgery, simulators for arthroscopic procedures, which require extensive practice to master, have been developed and shown to benefit clinical performance. This study aimed to evaluate the effectiveness of 2 types of arthroscopy simulators in medical education by assessing medical students without prior surgical training.

**Methods:**

We prospectively and randomly evaluated the effectiveness and satisfaction of arthroscopy simulators using 2 devices: a high-fidelity virtual reality simulator (ArthroSim) and a low-fidelity simulator (AZBOTS). To assess simulator effectiveness, 28 first-year medical students received 90 min of training on either device. Their knee arthroscopy skills, including tasks such as visualizing and probing the medial compartment and cruciate ligaments, were assessed using ArthroSim. Skill acquisition was measured by procedure completion time and the Arthroscopic Surgery Skill Evaluation Tool Global Rating Scale. Additionally, to assess trainee satisfaction, 109 fifth-year medical students completed a 7-point Likert scale questionnaire evaluating spatial judgment, hand–eye coordination, camera navigation, instrument handling, and the overall knee arthroscopy training experience.

**Results:**

No significant differences in skill acquisition were observed between the 2 simulator groups. Likewise, there were no significant differences in total questionnaire scores or in spatial judgment, hand–eye coordination, and camera navigation. However, participants using the high-fidelity simulator reported significantly greater satisfaction with instrument handling (*p* = 0.024) and the overall knee arthroscopy training experience (*p* = 0.033).

**Conclusions:**

Although skill acquisition did not differ significantly between the high- and low-fidelity simulators after a single training session, the high-fidelity simulator markedly improved trainee satisfaction, especially in instrument handling and perceived quality of the training experience. These findings suggest that simulator fidelity enhances the educational value of the arthroscopic training for novices emphasizing the role of realistic simulation in early surgical education.

## Introduction

Arthroscopic surgery is a widely performed procedure by orthopedic surgeons, valued for its low morbidity, diagnostic accuracy, and therapeutic efficacy. However, it is technically demanding and requires a high level of psychomotor proficiency. Surgeons must interpret a three-dimensional joint space through a two-dimensional camera image while skillfully manipulating instruments [1, 2]. Acquiring　these skills in the operating room (OR) can be time-intensive and may pose risks to patients, such as damage to chondral surfaces during the early stage of surgeon’s learning curve [1]. Nevertheless, it is essential for trainees to have opportunities to make non-critical errors early in training without compromising patient safety [3]. As a result, surgical skill development outside the OR is crucial, particularly for individuals new to surgery.

Over recent decades, evidence supporting simulation in medical education has expanded, highlighting its significance in surgical training. Multiple studies have recommended incorporating simulation into novice surgical training programs [4–7]. Arthroscopic simulators can be categorized into 3 types: physical models, virtual reality (VR) models, and VR–physical hybrid models. Physical models may include human or animal specimens or artificial benchtop simulators and are often evaluated based on fidelity, a measure of how closely the simulator replicates real-world surgical conditions. A key feature of VR simulation is haptic feedback, enabling trainees to experience tactile sensations. Additionally, VR simulators can provide performance feedback by simulating the level of force applied to joint surfaces.

Advances in simulator design have led to growing evidence supporting the improved transfer validity of arthroscopic simulation, particularly in enhancing intraoperative performance [8–12]. Although the integration of arthroscopic simulators into surgical training has become increasingly common, the relationship between simulator fidelity and its influence on skill acquisition and participant satisfaction in novice surgical education remains poorly understood.

The purpose of this study was to evaluate how simulator fidelity affects both skill acquisition and trainee satisfaction during arthroscopic knee surgery simulation. We hypothesized that a high-fidelity VR simulator would lead to greater improvements in arthroscopic skill development and higher satisfaction among trainees.

## Materials and methods

A prospective, randomized, comparative study was conducted using 2 types of simulators. The high-fidelity simulator was the ArthroSim VR simulator (ArthroSim), while the low-fidelity benchtop simulator was the AZBOTS. Participants were assigned to a simulator using the envelope method, in which each individual drew a card indicating one of 2 simulator types from sealed envelopes. Skill acquisition was evaluated in first-year medical students (*n* = 28), while satisfaction with the training experience was assessed in fifth-year medical students (*n* = 109) (Table 1). Written informed consent for participation in the study was obtained from all participants. This research was approved by the Institutional Review Board (IRB) of the authors’ affiliated institutions (IRB number: 1711011).

**Table 1:** Participant characteristics

	ArthroSim (high-fidelity)	AZBOTS (low-fidelity)	*P* values
*First-year medical student for skill acquisition*			
Number of participants	17	11	
Sex (female/male)	0/17	2/9	0.146†
*Fifth-year medical student for participants’ satisfaction*			
Number of participants	58	51	
Sex (female/male)	20/38	17/34	0.900‡

^†^Fisher’s exact test

^‡^Chi-square test

### Simulator 1. High-fidelity simulator; ArthroSim

**Fig. 1:**
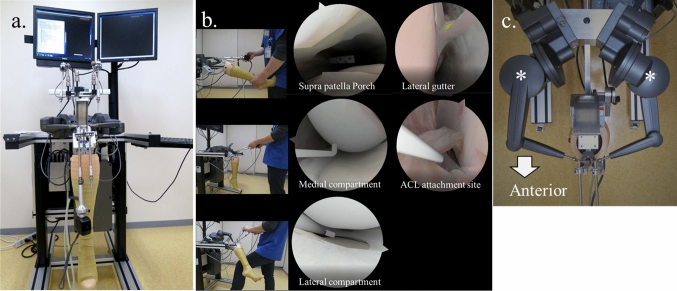
ArthroSim virtual reality knee simulator. a. Overall image of the simulator, b. High-resolution graphics and real-time graphical adjustments that respond to knee motion. ACL, anterior cruciate ligament. c. This simulator is equipped with 2 haptic devices, allowing participants to practice using both hands simultaneously. Asterisks indicate the haptic devises

The ArthroSim VR simulator (TolTech Touch of Life Technologies, Aurora, Colorado, USA; Fig. 1 [13, 14]) is a high-fidelity VR system that integrates 3 key features:1) High-resolution anatomical imaging developed using technology from the University of Colorado Life Science Center through the Visible Human Project, led by the US National Library of Medicine; 2) Real-time graphical adjustments in response to joint flexion, extension, and Varus/valgus stress, enabled by finite element modeling; and 3) A realistic tactile experience delivered by a high-resolution haptic feedback system. The simulator includes 2 haptic feedback devices, allowing users to simultaneously operate the camera and probe during training exercises. During the 90-min training session, the participants in the ArthroSim group followed a standardized curriculum for novices. The curriculum included basic camera navigation exercises. These exercises comprised: entering the joint via the anterolateral portal and systematically inspecting the suprapatellar pouch, patellofemoral joint, medial and lateral gutters, medial meniscus and femorotibial joint surface, anterior cruciate ligament, posterior cruciate ligament, and lateral meniscus and lateral femorotibial joint. The curriculum also involved fundamental probing exercises to familiarize the trainees with the essential psychomotor skills required for performing arthroscopy, such as systematically palpating the aforementioned structures to assess surface integrity and evaluating tension with a virtual probe.

### Simulator 2. Low-fidelity simulator; AZBOTS

**Fig. 2:**
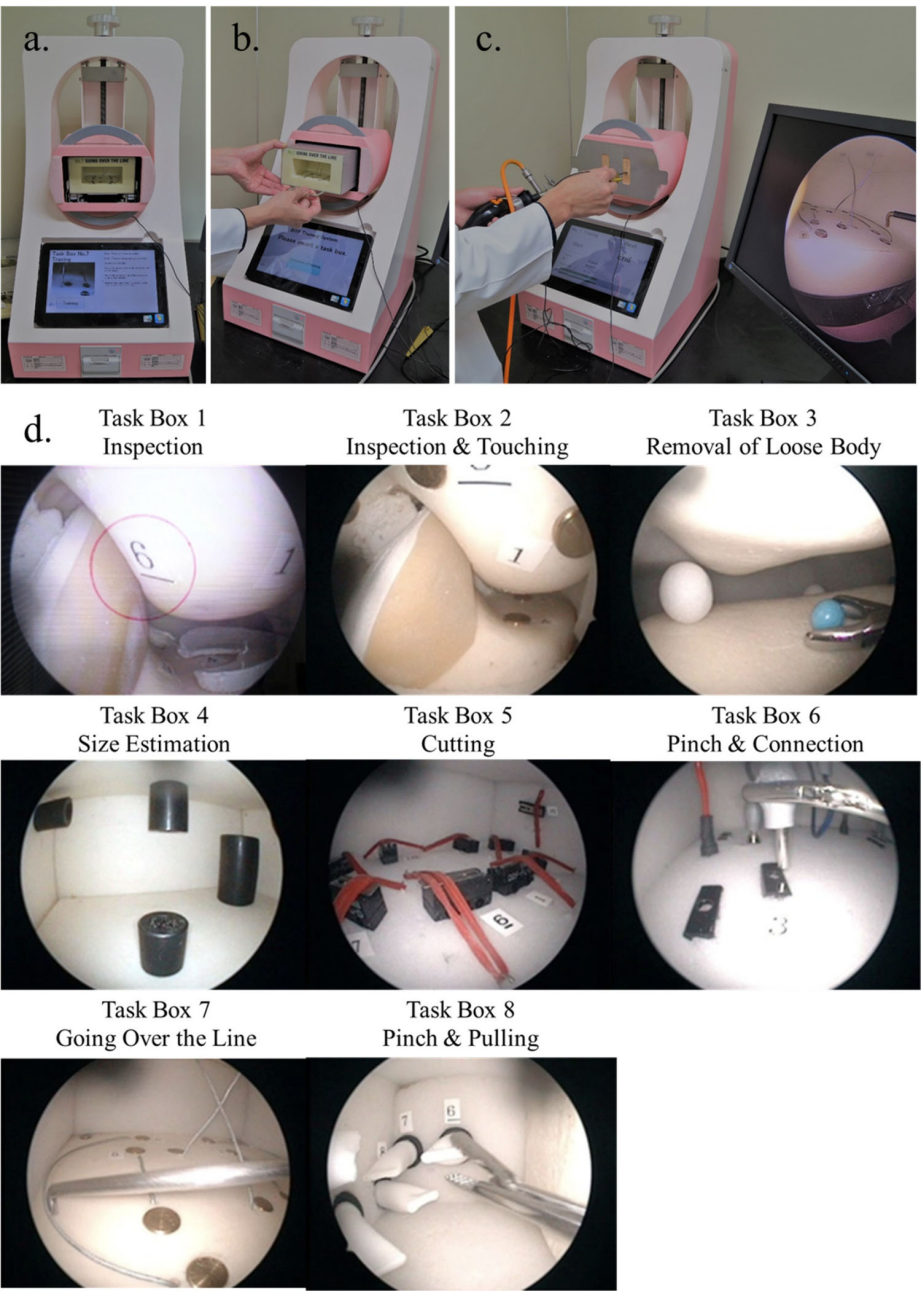
AZBOTS. The training program begins after the task box is inserted. a. Front cover is removed. b. The task box is inserted. c. Actual training in progress. d. 8 tasks included in the box training device for basic arthroscopic surgical skill development

The AZBOTS simulator (Marui & Co., Ltd., Osaka, Japan; Fig. 2) is a low-fidelity device developed for training surgeons in various arthroscopic procedures. The simulator includes 8 task boxes, each designed to simulate different components of the surgical process:

- Task Box 1: Inspection: participants search for numerals in sequential order, beginning with “1,” and proceed to the next number by pressing a foot switch.
- Task Box 2: Inspection and touching: participants touch a probe to metal disks.
- Task Box 3: Removal of loose body: participants use forceps to remove a loose body (a Styrofoam ball, representing a free body) from a nylon protrusion.
- Task Box 4: Size estimation: participants use a probe to touch cylindrical objects in order from smallest to the largest.
- Task Box 5: Cutting: participants cut rubber bands using scissors.
- Task Box 6: Pinch and connection: participants remove a jack from one hole and insert it into another.
- Task Box 7: Going over the line: participants use the probe to touch an initial metal disk and trace a guide wire to the terminal disk.
- Task Box 8: Pinch and pulling: participants use forceps Trainees to pinch and pull out a jack.

Training is guided by prompts such as time limits for task completion and auditory alarms to signal errors. Performance can be quantitatively assessed using completion scores, displayed after each task. The use of actual surgical instruments during training tasks enables participants to develop proficiency in arthroscopic tool handling. During the 90-min training session, the participants in the AZBOTS group were instructed to complete the 8-task boxes in a sequential order, with repeating the tasks as time allowed, to practice fundamental skills such as inspection, instrument handling, and object manipulation.

### Skill and satisfaction assessment

**Fig. 3:**
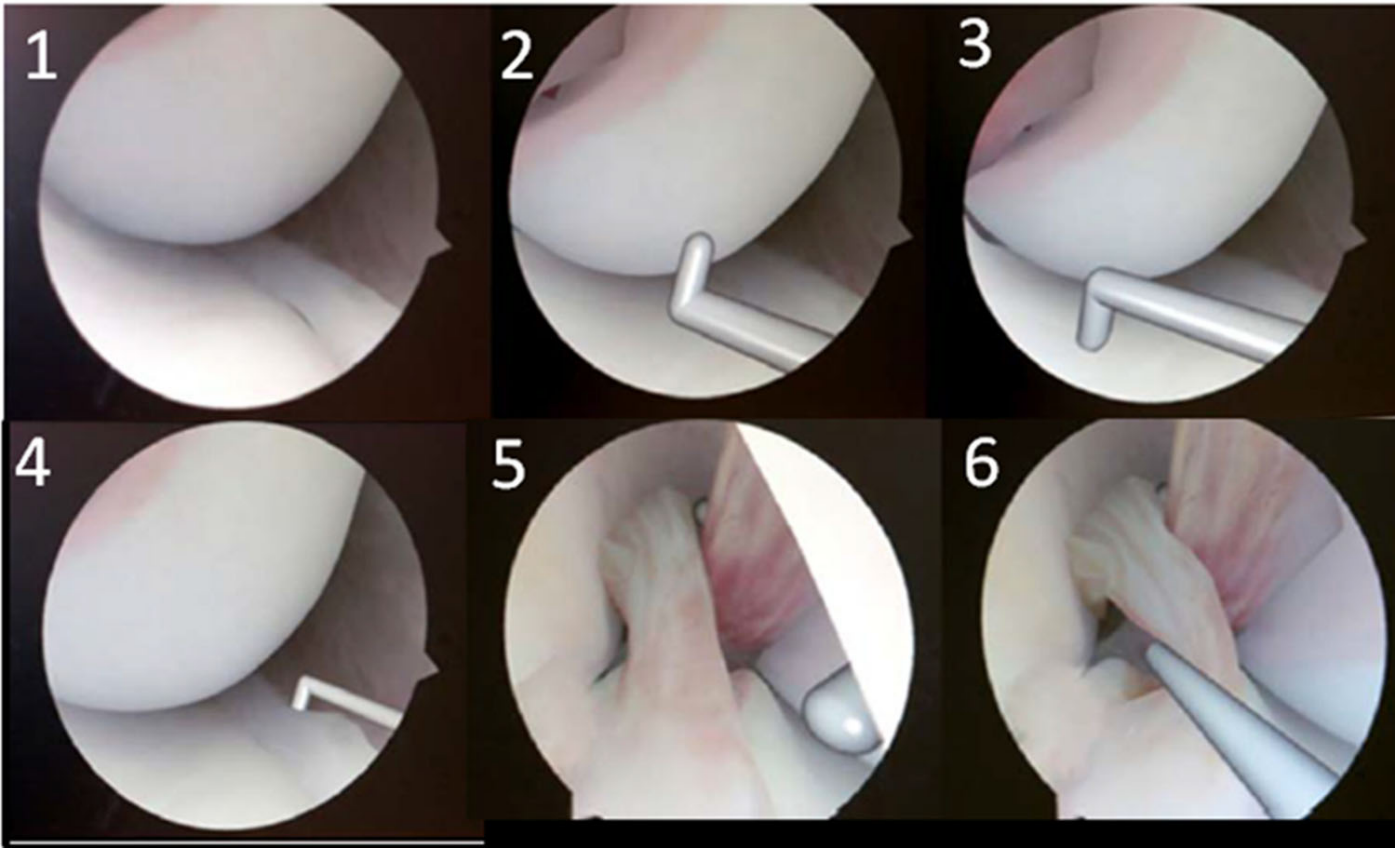
A series of tasks for assessment using ArthroSim after training. 1, observing the medial compartment; 2, probing the femoral cartilage; 3, probing the tibial cartilage; 4, observing and probing the medial meniscus; 5, observing the intercondylar notch; 6, observing and probing anterior and posterior cruciate ligaments

Following training, participants were evaluated for knee arthroscopy skills using ArthroSim. The assessment involved a series of tasks, including observing and probing the medial compartment (femoral and tibial articular surfaces and medial meniscus), anterior cruciate ligament, and posterior cruciate ligament, performed 3 times (Fig. 3). To ensure that the assessment measured procedural skill rather than basic familiarity with the ArthroSim, all participants underwent a brief orientation session on the ArthroSim simulator prior to the evaluation. This session aimed to comprehensively orient the trainees with the fundamental principles of camera and instrument manipulation, focusing on the essentials of the specific assessment tasks.

Skill acquisition was measured based on the time required to complete the tasks and scores from the Arthroscopic Surgery Skill Evaluation Tool Global Rating Scale (ASSET Score), which assesses technical proficiency across multiple domains; procedure safety, field of view, camera and instrument dexterity, bimanual dexterity, and flow of procedure [15]. The mean values from the 3 assessments were used for evaluation.

Trainee satisfaction was measured using a questionnaire the evaluated 5 aspects of the training experience: spatial judgment, hand–eye coordination, camera navigation, handling of surgical instrument, and usefulness of knee arthroscopy training. Responses were recorded on a 7-point Likert scale (1: strongly disagree, 2: disagree, 3: slightly disagree, 4: neither agree nor disagree, 5: slightly agree, 6: agree, 7: fully agree) [16–18]. Additionally, all participants responded to 2 statements regarding their training experience with the arthroscopic simulator, with response options of “Agree,” “Disagree,” or “Undecided”. The statements were: (1) simulator training should be conducted before actual surgery for beginners with no arthroscopic experience; and (2) would you recommend arthroscopy simulator training to colleagues?

### Statistical analysis

The normality of the data distribution was assessed using the Shapiro–Wilk normality test. For composite scores, the time taken to complete the tasks that were normally distributed is presented as the mean, standard deviation, and 95% confidence interval, while nonnormally distributed components are reported as the median and interquartile range. Frequencies are expressed as percentages (%). A sample size analysis was conducted using G*Power software (version 3.1) to assess the statistical power of the study. The analysis confirmed that the sample size was sufficient to detect a significant difference between groups. An improvement of 2 points in the ASSET score was considered at relevant difference. A total of 28 participants (ArthroSim: 17 participants, AZBOTS: 11 participants) were needed to detect a mean difference of 1.0 ± 0.8 points in the ASSET score with a two-sided 5% significance level and 80% power. All statistical analyses were performed using JMP software version 14 (SAS Institute Inc., Cary, North Carolina, USA). Student’s t-test was used to analyze normally distributed data with homogeneity of variance verified using the Levene test. The nonparametric Wilcoxon rank-sum test applied to analyze data that did not meet normality assumptions. Categorical variables are presented as frequencies and percentages and were compared using Fisher’s exact test and the Chi-square test. All statistical tests were two-sided, and a p value of < 0.05 was considered statistically significant.

## Results

No significant differences in skill acquisition were observed between the 2 simulator groups, as measured by the time taken to complete the tasks and both total ASSET scores and individual component scores (Table 2). Similarly, no significant differences were found in total questionnaire scores or in aspects of spatial judgment, hand–eye coordination, and camera navigation (Table 3). However, participants using ArthroSim (high-fidelity simulator) reported significantly higher satisfaction in handling of surgical instruments (*p* = 0.024) and training of knee arthroscopy (*p* = 0.033) compared to those using AZBOTS (low-fidelity simulator) (Table 3). Over 96% of participants agreed with statement, "Beginners with no experience of arthroscopic surgery should undergo training using simulators before actual surgery," and over 94% agreed with the statement, "I would recommend surgical training using arthroscopic simulators to my colleagues" (Table 4). Importantly, no participants disagreed with either of these statements (Table 4).

**Table 2:** Comparison of skill acquisition evaluations between high-fidelity and low-fidelity simulators

	ArthroSim (high-fidelity) *N* = 17	AZBOTS (low-fidelity) *N* = 11	*P* values
Time taken to complete the tasks (seconds) Mean (SD, 95%CI)	49.5 (11.9, 43.4 to 55.6)	47.9 (15.3, 37.6–58.2)	0.617†
Total ASSET Score (points, maximum 30) Median (IQR)	12.7 (11.3–17.5)	13.3 (13.0–15.3)	0.509‡
1. Procedure safety (points) Median (IQR)	2.7 (2.0–3.3)	2.7 (2.0–3.3)	0.721‡
2. Field of view (points) Median (IQR)	2.3 (2.2–2.8)	2.7 (2.0–2.7)	0.514‡
3. Camera dexterity (points) Median (IQR)	2.0 (1.8–2.7)	2.3 (1.7–2.3)	0.924‡
4. Instrument dexterity (points) Median (IQR)	2.0 (2.0–3.2)	2.3 (1.7–2.7)	0.962‡
5. Bimanual dexterity (points) Median (IQR)	1.3 (1.0–1.3)	1.6 (1.7–2.3)	0.121‡
6. Flow of procedure (points) Median (IQR)	2.3 (1.7–3.0)	2.3 (2.0–2.7)	0.981‡

Evaluation was based on six items in the ASSET score, each with a maximum value of 5. ASSET score, Arthroscopic Surgery Skill Evaluation Tool Global Rating Scale [11]

^†^Student’s t test

^‡^Wilcoxon rank-sum test

**Table 3:** Comparison of participant satisfaction between simulators (questionnaire)

	ArthroSim (high-fidelity) *N* = 58	AZBOTS (low-fidelity) *N* = 51	*P* values
Total Score (points, maximum 35) Median (mean) (IQR)	35 (35) [33–35]	34 (33) [31–35]	0.085
1. Spatial judgement (points) Median (mean) [IQR]	7 (6.7) [6–7]	7 (6.7) [6–7]	0.930
2. Hand–eye coordination (points) Median (mean) [IQR]	7 (6.7) [6–7]	7 (6.7) [6–7]	0.923
3. Camera navigation (points) Median (mean) [IQR]	7 (6.7) [6–7]	7 (6.5) [6–7]	0.245
4. Handling of surgical instrument (points) Median (mean) [IQR]	7 (6.7) [6–7]	7 (6.5) [6–7]	0.024*
5. Usefulness of knee arthroscopy training (points) Median (mean) [IQR]	7 (6.8) [7–7]	7 (6.5) [6–7]	0.033*

Evaluation was based on five items in the questionnaire, each with a maximum value of 7. Asterisks represent statistical significance with Wilcoxon rank-sum test

**Table 4:** Comparison of participant satisfaction between simulators (questionnaire)

	ArthroSim (high-fidelity) *N* = 58	AZBOTS (low-fidelity) *N* = 51
*Beginners without experience in arthroscopic experience: Simulator training should be conducted before training in actual surgery (%)*		
Agree	96.6	98.0
Undecided	3.4	2.0
Disagree	0.0	0.0
*Recommend arthroscopy simulator training to colleagues (%)*		
Agree	94.8	94.1
Undecided	5.2	5.9
Disagree	0.0	0.0

## Discussion

The most important finding of this study is that a single training session using the high-fidelity simulator, ArthroSim, did not result in statistically significant differences in skill acquisition compared to the low-fidelity simulator, AZBOTS. Notably, this lack of difference was found despite the potential advantage in the ArthroSim group. This finding underscores the effectiveness of AZBOTS in teaching essential arthroscopic skills to novices. However, ArthroSim significantly enhanced participant satisfaction, particularly in instrument handling and the overall knee arthroscopy training experience. These findings suggest a positive influence of higher simulator fidelity on trainee satisfaction, which may be important for enhancing motivation and potentially supporting long-term skill development.

Since simulator-based training is widely accepted, prior studies have primarily emphasized objective performance outcomes, such as procedure time and instrument path efficiency [19–21]. However, these studies overlooked the subjective experiences that might enhance learning. Training satisfaction is a critical determinant of learning motivation and engagement, ultimately influencing the effectiveness of surgical training, specifically in novice trainees who may find it demanding [22, 23]. A positive training experience can serve as a strong motivator for continuing learning and encouraging more active participation in training and potentially lead to long-term improvements in surgical competence.

Although reports directly evaluating how simulator fidelity impacts participant satisfaction are scarce [7, 24], some studies have indicated higher engagement with VR simulators compared with the bench-top models [25]. Our study contributes specific evidence in this area, demonstrating that the high-fidelity simulator was superior in enhancing satisfaction for tasks involving "instrument handling" and "the overall knee arthroscopy training experience." This can be attributed to the immersive surgical environment created by the VR simulator. The anatomically precise graphics and haptic feedback of VR simulator provide a more realistic and authentic training experience [26]. This immersive realism is consistent with the cognitive load theory, which suggests that well-designed, high-fidelity simulators help learners manage intrinsic cognitive load, thereby improving their ability to process and retain new information [27, 28]. Consequently, simulator fidelity influences not only technical skill development but also the crucial psychological and motivational dimensions of learning.

This study has some limitations. First, it evaluates only the short-term effects of a single training session, and the long-term impact of repeated training on skill improvement remains unclear. Future studies should explore the effects of multiple sessions, including skill retention and long-term learning outcomes. Second, the study population consisted solely of novice participants without surgical experience, and the effects of simulator fidelity on experienced surgeons were not evaluated. Future research should include participants with various levels of surgical expertise to assess the broader applicability of VR simulation in surgical education. Third, this study compared between only 2 types of simulators (ArthroSim and AZBOTS); other simulation modalities or training methods were not assessed. Furthermore, since this study focused on knee arthroscopy, the generalizability of the findings to other surgical procedures requires further investigation. However, the principle that simulator fidelity influences learner satisfaction and motivation might be applicable to other domains of surgical simulation. Further research comparing diverse simulation tools and instructional approaches would be valuable for optimizing training curricula. Fourth, skill in both groups was assessed exclusively by the ArthroSim simulator. This might have introduced a potential bias favoring the group that trained using the same device. Nevertheless, novices using the low-fidelity AZBOTS simulator acquired the same level of arthroscopic skill as did those using the high-fidelity ArthroSim simulator. This suggests that the low-fidelity simulator is important for training novices in knee arthroscopic procedures at an early stage. Future studies should consider using neutral assessment methods or evaluating skill transfer to a different platform, cadaveric model, or animal model to mitigate this potential bias. Additionally, future studies could explore how specific simulator features, such as adaptive difficulty levels and real-time feedback, influence learning outcomes to better inform the design of simulation-based training.

In conclusion, this study demonstrated that in knee arthroscopy training for novice participants, high-fidelity simulators significantly improve training satisfaction compared to low-fidelity simulators. This difference was evident after only a single training session, highlighting the immediate influence of simulator fidelity on the trainees’ subjective experience. These findings suggest that simulator fidelity in surgical training influences not only technical skill development but also learner satisfaction. High-fidelity simulators may offer advantages in early stage training by providing a more immersive and engaging learning experience. The development of more cost-effective high-fidelity simulators will be essential for expanding access to high-quality surgical training. The findings of this study contribute to the advancement of knee arthroscopy simulation training and the overall improvement of surgical education quality. Ultimately, structured integration of simulation into surgical curricula, supported by further research, will be essential in shaping the future of surgical education and enhancing patient safety.
